# Meat-egg-dairy consumption and depressive symptoms among Chinese older adults: exploring rural/urban and gender disparities

**DOI:** 10.3389/fpsyt.2024.1489387

**Published:** 2024-11-26

**Authors:** Boyu Si, Keqing Zhang

**Affiliations:** School of English and International Studies, Beijing Foreign Studies University, Beijing, China

**Keywords:** depressive symptom, MED consumption, depression, Chinese longitudinal healthy longevity survey, rural/urban disparities, gender differences

## Abstract

**Introduction:**

This study examines the association between Meat, Egg, and Dairy (MED) product consumption and depressive symptoms among older adults in China, focusing on rural/urban and gender differences.

**Methods:**

This study employed data from the latest wave (year 2018) of the Chinese Longitudinal Healthy Longevity Survey (CLHLS), and Ordinary Least Squares (OLS) models were applied to examine the association between MED consumption and depression levels.

**Findings:**

The findings revealed a consistent negative relationship between MED consumption and depression, with higher MED intake associated with lower depression levels. Urban residents and males exhibited higher MED consumption, which correlated with less depressive symptoms. However, the impact of MED consumption on depression varied by subgroups; urban older adults benefited more from MED consumption than their rural counterparts, and the relationship between MED intake and depression was more pronounced in males than in females. The study highlighted the influence of socio-demographic factors, such as literacy, income, and self-rated health, on depression levels.

**Discussion:**

The results suggested that MED consumption may offer protective effects on mental health in older adults, although the association may not be causal. The study underscored the need for further research to explore the complex interplay between diet and mental health in older adults, particularly in diverse cultural contexts.

## 1 Introduction

The dietary patterns of older adults correlate with their physical and mental health condition, particularly in the context of depressive symptoms, which represent a state of mental, emotional, and social health of an individual, which is a somewhat malleable concept to do with people’s feelings about their everyday-life activities and represents a psychological state with positive functioning and the absence of mental illnesses (1, 2). With the global population aging, comprehending the complex interplay between dietary patterns and the depressive symptom is becoming increasingly crucial for promoting healthy aging and mitigating the burden of age-related health issues. Mental health is essential for individuals, providing the vitality necessary for active living, goal achievement, and social interaction. Mental health, characterized by cognitive, emotional, and behavioral dysfunctions, significantly disrupts personal life and productivity. It is likely to impair older adults’ capacity to perform fundamental daily activities, diminishing their independence, autonomy, and quality of life, while also contributing to their isolation, loss of independence, loneliness, and psychological distress, as a risky factor for depression, dementia and Alzheimer’s Disease (3–6). One previous cross-sectional study revealed that among the elderly Chinese primary care patients, 30.6% had depression, which correlated to the findings from meta-analyses of population-based studies reporting that 23.6% and 2.7% of the Chinese elderly patients had depressive symptoms and major depressive disorder respectively (7). While the depressive symptom of older adults is influenced by a range of factors, including education level, poor financial status, lack of an exercise habit, chronic medical conditions, and loneliness (8). Recent research has underscored the essential impact of dietary factors on depressive symptoms (9, 10). Consequently, elucidating the effects of specific dietary components on depressive symptoms has become a significant area of widespread interest for researchers and healthcare practitioners.

Compared to other dietary components, a relative paucity of research has examined the association between the consumption of meat, eggs, and dairy (MED, as *Rou-Dan-Nai* in Chinese) products and depressive symptoms among older adults. Recommended by the Food and Agriculture Organization of the United Nations (FAO), meat, eggs, and dairy products offer crucial sources of much-needed nutrients that cannot easily be obtained from plant-based foods. They play a vital role in preventing diseases and sustaining energy, which is closely related to human well-being (11). Previous studies have demonstrated that protein intake is cross-sectionally associated with visuospatial, verbal fluency, processing speed, sustained attention, immune function, and recuperation from illness in older adults (12–14). However, excessive consumption of certain MED components, such as milk and dairy products, has been associated with negative health outcomes, encompassing overweight, obesity, hypertension, and hyperlipidemia (15–17). Much research in recent years has focused on the influence of MED consumption on the physical health of older adults, while few researchers have addressed the relationship between MED consumption and depressive symptoms among older adults, and this study aims to describe and examine such associations.

The MED-Depression relationship holds particular significance in China, given the rapidly increasing elderly population and evolving dietary habits. The social and economic experiences throughout their lives continue to shape the food attitudes and practices of older Chinese adults (18). In recent decades, China’s national dietary patterns have undergone a significant transformation, marked by a shift towards ‘Westernization’, characterized by a decreased intake of plant foods and an increased intake of animal foods (19). For an extended period, both the Chinese government and its citizens have been closely concerned about MED consumption (20, 21). It is essential to elucidate the time-varying changes in MED consumption and the corresponding levels of depressive symptoms among older adults. Given China’s rapidly aging population and increasing prevalence of depression among older adults, understanding the relationship between commonly consumed foods like MED and mental health could inform public health policies and preventive strategies. Meanwhile, healthcare providers need evidence-based guidelines for dietary recommendations, especially for vulnerable populations like older adults. Comprehensive research on MED consumption could inform more targeted nutritional interventions for mental health management.

In addition, the relationship between MED and depressive symptoms may vary significantly based on key demographic factors, with *Hukou* System (household Registration) status being particularly influential. The *Hukou* system significantly shapes Chinese citizens’ life chances and migratory behavior by recording resident identities within specific zones and restricting settlement through urban quotas. Beyond mere registration, it limits access to migration by controlling the transfer of *Hukou* residency to urban areas (22). A discernible disparity in dietary patterns exists between rural and urban populations in China. Rural inhabitants demonstrate a propensity towards plant-centric nutritional habits and reduced consumption of animal-derived products, while urban citizens hold the contrary dietary pattern, suggesting a lower MED consumption correlated to more depressive symptoms among rural residents compared to their urban counterparts (23, 24). In addition to the rural-urban disparities, gender influences on MED-Depression emerge as a crucial variable within the Chinese context. As an illustration, previous investigations have indicated that elderly female demographics in China exhibit a propensity for reduced consumption of animal-derived nutritional sources, encompassing meat, eggs, and dairy products, relative to their male counterparts (25, 26). Consequently, the *Hukou* & gender-based dietary differentiation underscores the necessity of considering both geographical and gender-related factors in analyses of the association of Chinese MED-Depression among older adults.

## 2 Background

### 2.1 Dietary patterns and the depressive symptom

The relationship between dietary patterns and depressive symptoms has gained significant attention in recent years, particularly in the context of aging populations. The relationship between diet and mental health is complex, influenced by various factors such as nutrient composition, food quality, and cultural conventions. As the global population ages, their nutritional needs and eating habits may change, profoundly affecting both physical and mental health outcomes. Understanding these relationships is crucial for developing effective strategies to promote ‘happy and healthy aging’ and improve the quality of life for older adults.

Research has consistently highlighted the relationship between specific dietary patterns and various aspects of mental health, such as depression and cognitive function. For instance, adherence to the Mediterranean diet, characterized by a high intake of fruits, vegetables, whole grains, fish, and olive oil, has been consistently associated with better mental health outcomes. A systematic review by Martínez-González et al. (27) found that some aspects of the traditional Mediterranean diet might have contributed to lowering the risk of depression. Conversely, Western dietary patterns, characterized by high consumption of red and processed meats, refined grains, sugary beverages, and high-fat dairy products, are linked to poorer mental health outcomes, including depressive symptoms, anxiety, and cognitive decline (28, 29). Other dietary patterns, including Dietary Approaches to Stop Hypertension (DASH), the Traditional Chinese dietary pattern, and the sugar-rich dietary pattern, are recognized as significant contributors to the mental health of older adults (30, 31).

Despite valuable insights from existing literature on the relationship between overall dietary patterns and depressive symptoms in older adults, there remains a significant gap in understanding the impact of specific dietary components, particularly individual food groups like meat, eggs, and dairy products. Since foods are rarely consumed in isolation, and their nutrients may interact synergistically, potentially amplifying or moderating their effects on mental health. Understanding these interactions could lead to more effective dietary recommendations for depression prevention. Further research into these specific elements is both necessary and currently underexplored.

### 2.2 Meat, egg, and dairy product consumption and the depressive symptom

The relationship between meat, egg, and dairy product consumption (MED) and the depressive symptom has been a subject of increasing interest in nutritional research. Given that meat, eggs, and dairy products are significant sources of protein, it is worth considering the broader role of protein intake in mental health. It is established that adequate protein intake is crucial for maintaining brain health and may play a role in preventing age-related cognitive decline (32).

Despite the limited number of studies directly investigating the relationship between MED consumption and depressive symptoms, some research indicates that certain MED components may offer potential benefits for mental health. For instance, Dobersek et al. (33) investigates the relationship between meat consumption, depression, and anxiety, indicating that individuals who consume meat exhibited lower rates of depression and anxiety compared to those who abstain from meat, which implies meat consumption might confer protective benefits for mental health. Simultaneously, some studies have found a relationship between egg consumption and depressive symptoms in the elderly, suggesting that individuals consuming a certain amount of eggs per week have lower odds of depression compared to those who do not consume eggs (34, 35). Moreover, dairy products, particularly those low in fat, are proven to have potential mental health benefits associated with a lower risk of cognitive decline in older adults (36–38).

The relationship between the consumption of meat, eggs, and dairy products (MED) and depressive symptoms has been a focal point of recent research, due to the essential nutrients these foods provide, such as high-quality proteins, vitamins, and minerals, which are crucial for brain health and cognitive function (39, 40). A longitudinal study by Zhang and Wu (41) investigates the association between MED consumption and frailty, which is a crucial factor in older adults’ physical health, while their mental health condition remaines unclear. The link between MED consumption and mental health is complex, influenced by multiple factors, and has not been comprehensively studied as a collective group of foods. To explore the psychological effects of MED consumption, it is necessary to conduct a thorough analysis that considers the interactions between various dietary components.

However, it’s important to note that the relationship between MED consumption and health outcomes, including mental health, is not uniformly positive. Several studies have highlighted potential concerns. For instance, previous research finds that processed meat consumption is associated with an increased risk of depressive symptoms, potentially through inflammatory pathways, which indicates the potential links between pro-inflammatory diets and depression risk (42, 43). Regarding dairy consumption, it is reported that high milk intake might not always confer health benefits, particularly when considering long-term health outcomes (44). The complexity extends to egg consumption, where Zhong et al. (45) observe that higher dietary cholesterol intake (including from eggs) is significantly associated with increased risk of cardiovascular disease and mortality, conditions often comorbid with depression in older adults. These findings underscore the importance of considering both the quantity and quality of MED consumption, as well as individual differences in metabolic responses. This study aims to address this gap by examining the combined effects of MED consumption on depressive symptoms through a comprehensive approach.

### 2.3 The context of China: disparities by *Hukou* status and gender

To fully understand the relationship between meat, egg, and dairy (MED) consumption and depressive symptoms among older Chinese adults, it is crucial to consider the unique context of China, particularly the disparities that exist based on *Hukou* status (rural/urban residence) and gender. These factors significantly influence dietary patterns, food access, and overall depressive symptoms in the Chinese population.

The *Hukou* system, China’s household registration system, has created significant socioeconomic disparities between rural and urban areas. The *Hukou* system, established in the 1950s as a social control mechanism during China’s planned economy era, has created profound socioeconomic divisions between rural and urban populations that persist today (46). This household registration system initially restricts population mobility and resource allocation, leading to systematic differences in education, healthcare, and dietary resources between rural and urban areas (23). Recent data shows that urban residents’ annual disposable income averages ¥47,122, more than 2.5 times higher than rural residents’ ¥18,748, directly impacting their food purchasing power and dietary choices (47). These disparities extend to dietary patterns, food consumption, and access to nutrition (48). The nutrition transition in China is characterized by shifts in dietary patterns associated with rapid economic development and urbanization. This dietary transition has significant health consequences, which might include impacts on mental health and well-being, particularly among older adults who have experienced these changes over their lifetimes. Urban areas in China have greater access to diverse food options, including MED products, while rural residents are more likely to adhere to traditional dietary patterns that are lower in meat, eggs, and dairy products. The nutrition transition in China, accelerated by rapid economic development and urbanization, has led to marked dietary disparities. Urban residents consume significantly more animal products, showing that the proportion of energy provided by protein and fat intake for urban residents was 12.9% and 36.4% in 2015 compared with 11.5% and 33.2% for rural residents, respectively (49). This dietary transition has significant health consequences, potentially impacting mental health and well-being, particularly among older adults who have experienced these changes over their lifetimes. Research indicates that urban elderly have 22% higher protein intake compared to their rural counterparts (50). This rural-urban divide in food environments could potentially influence the depressive symptoms of older adults through differences in nutritional intake (51–54).

While the *Hukou* system creates geographical disparities in food access and consumption patterns, gender introduces another layer of inequality in Chinese society, particularly affecting dietary habits and mental health outcomes. This intersection of residential status and gender creates unique vulnerabilities in the Chinese population. Gender disparities in MED consumption and depressive symptom outcomes might also be significant in China. Traditionally, Chinese dietary practices favored men, with women often consuming fewer animal-based foods and more plant-based foods. This gender-based dietary pattern had implications for mental health, as women were more likely to suffer from nutritional deficiencies that could contribute to mental health disorders (55, 56). Contemporary studies show that women consume approximately 16.39% less protein and 12.67% less fat compared to men across all age groups (57). This gender-based dietary pattern might have implications for mental health, as women show higher rates of nutritional deficiencies that could contribute to mental health disorders.

The relationship between dietary patterns, particularly MED consumption, and depressive symptoms among older adults in China is shaped by a complex interplay of cultural, economic, and social factors. Understanding these disparities is crucial for developing targeted interventions that promote mental health and well-being among older adults in China.

## 3 Method

### 3.1 Data

This study employed data from the Chinese Longitudinal Healthy Longevity Survey (CLHLS), which was initiated to explore the determinants of healthy longevity among the elderly population in China and to enhance our understanding of the aging process. The survey, initially conducted across 22 provinces, adopted a multi-stage, non-equal target random sampling method. In an attempt to ensure a sufficient sample of Chinese older adults, individuals aged 80 and above were oversampled. Data were collected through face-to-face interviews by trained interviewers. The study was approved by the Ethical Review Committee of Peking University, and written informed consent (IRB00001052–24713074) was obtained from all participants.

### 3.2 Study sample

To ensure the reliability of the model, this study utilized data from the latest wave (year 2018), exclusively focusing on community-dwelling individuals aged 55 years and older. Meanwhile, with some modifications, respondents lacking information on MED consumption or depression were excluded, resulting in a final sample of 1,937 participants. To address any missing data on demographic variables and other covariates, multiple imputations were performed.

### 3.3 Measures

#### 3.3.1 Dependent variable

The depressive symptom was measured with depression (58, 59). The Center for Epidemiologic Studies Depression Scale (CES-D-10) was utilized to measure depressive symptoms due to its extensive validation and reliability in assessing mental health among older adults (60). Respondents were asked to rate how they have felt and behaved during the past week over ten items: “I was bothered by things that don’t usually bother me,” “I had trouble keeping my mind on what I was doing,” “I felt depressed,” “I felt everything I did was an effort,” “I felt hopeful about the future,” “I felt fearful,” “My sleep was sleepless,” “I was happy,” “I felt lonely,” and “I could not get going.” Responses ranged from “1. always” to “5. never”. All items except Item 5 (hopeful) and 8 (Happy) were reversely coded, and the average index was computed with a higher score reflecting higher levels of depression (1–5). The Cronbach’s alpha was 0.811, indicating good consistency.

#### 3.3.2 Main independent variable

To analyze the patterns of meat, egg, and dairy (MED) consumption among Chinese older adults, three dummy variables were constructed to indicate whether an individual regularly consumed meat, eggs, or dairy products. Consumption frequency was evaluated in accordance with the China Nutrition Guidelines. Individuals who consumed MED daily or several times per week were assigned a value of 1 (yes); conversely, those who did not were assigned a value of 0 (no). Given that some older adults may adhere to partial vegetarianism, fish consumption was also considered; if an individual consumed either fish or meat, the corresponding dummy variable was coded as 1. The overall MED consumption score, ranging from 0 to 3, was computed by summing the scores for the three items, with higher scores reflecting greater intake of MED products. This scoring system is henceforth referred to as 0-/1-/2-/3-MED consumption.

#### 3.3.3 Covariates

The sociodemographic covariates included in the analysis were age (measured in years), gender (male = 1), marital status (married/partnered = 1), *Hukou* status (rural = 1), educational attainment (literate = 1), sufficient income to cover daily expenses (yes = 1), and social insurance status (under social security or commercial insurance = 1). Additionally, health-related and behavioral controls encompassed smoking status (current/past smoker = 1), alcohol consumption (current/past drinker = 1), frequency of exercise (daily/several times per week = 1), life satisfaction levels (1-5, with higher scores indicating greater satisfaction), self-rated health (1-5, with higher scores reflecting better-perceived health), and the number of chronic diseases (0-12, with higher scores indicating a greater number of chronic conditions).

### 3.4 Analysis

**Figure 1:**
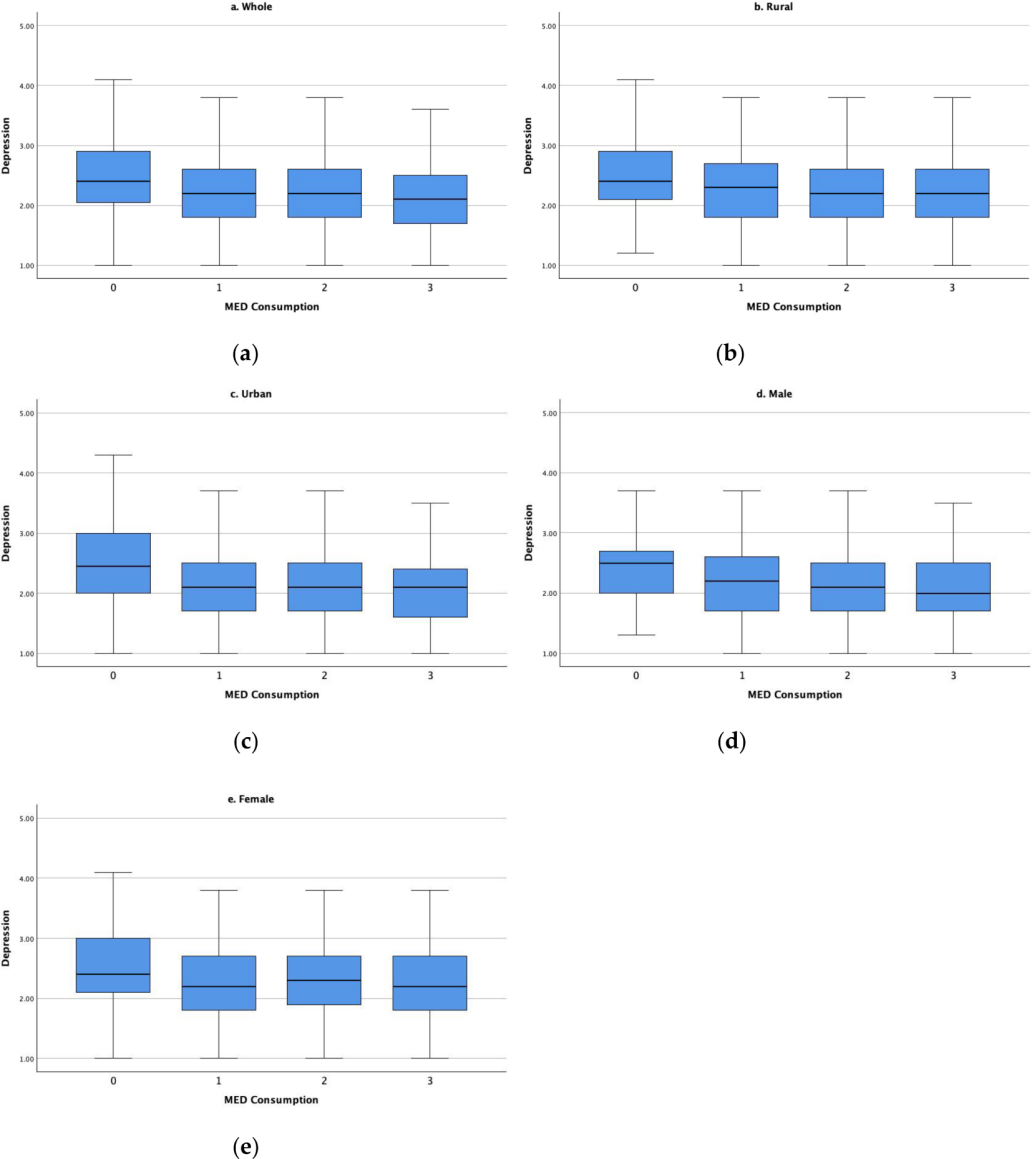
Depression levels by MED consumption **(A)** whole sample; **(B)** rural subsample; **(C)** urban subsample; **(D)** male subsample; **(E)** female subsample.

Data analysis was performed using RStudio, with descriptive statistics presented in **Table 1**. Paired t-tests and ANOVA tests were carried out to check the rural/urban as well as gender differences across all variables. **Figure 1** visualized the mean depression for older adults from different MED groups: 1a was about the whole sample, while 1b-1e showed subsample results. To examine the association between MED consumption and depression levels, Ordinary Least Squares (OLS) models were employed. Results were presented in **Tables 2**, **3**, controlling for covariates. **Table 2** showed the results for the whole sample, including the focal IV and covariates separately and together, while **Table 3** showed the results for the subsamples.

**Table 1:** Sample characteristics (Year 2018).

	Whole	Rural	Urban	Male	Female
	(*N*=1,937)	(*N*=1301)	(*N*=636)	(*N*=954)	(*N*=983)
*Main IV*	N (%)				
MED Consumption					
0	159 (8.21)	105 (8.07)	54 (8.49) ***	65 (6.81)	94 (9.56) **
1	496 (25.61)	364 (27.98)	132 (20.75)	224 (23.48)	272 (27.67)
2	936 (48.32)	634 (48.73)	302 (47.48)	476 (49.90)	460 (46.80)
3	346 (17.86)	198 (15.22)	148 (23.27)	189 (19.81)	157 (15.97)
*Categorical*	N (%)				
Gender: Male	954 (49.25)	648 (49.81)	306 (48.11) *		
*Hukou*: Rural	1301 (67.17)			648 (67.92)	653 (66.43) *
Married/Partnered: Yes	886 (45.74)	590 (45.35)	296 (46.54)	591 (61.95)	295 (30.01) ***
Literate: Yes	1075 (55.50)	677 (52.04)	398 (62.58) ***	716 (75.05)	359 (36.52) ***
Smoking: Yes	471 (24.32)	332 (25.52)	139 (21.86) *	412 (43.19)	59 (6.00) ***
Drinking: Yes	459 (23.70)	324 (24.90)	135 (21.23) *	370 (38.78)	89 (9.05) ***
Exercise: Yes	697 (35.98)	339 (26.06)	358 (56.29) ***	392 (41.09)	305 (31.03) ***
Social insurance: Yes	605 (31.23)	428 (32.90)	177 (27.83) *	294 (30.82)	311 (31.64)
Income: Sufficient	1669 (86.16)	1093 (84.01)	576 (90.57) ***	819 (85.85)	850 (86.47)
*Continuous*	Mean (SD)				
Age	84.15 (7.66)	83.91 (7.65)	84.64 (7.65) ***	83.45 (7.16)	84.83 (8.06) ***
Life Satisfaction	3.65 (0.81)	3.58 (0.81)	3.8 (0.79)	3.68 (0.79)	3.62 (0.83)
Self-Rated Health	3.59 (0.92)	3.54 (0.93)	3.70 (0.89) **	3.66 (0.91)	3.53 (0.93) **
Number of Chronic Diseases	1.5 (1.61)	1.37 (1.50)	1.76 (1.77)	1.45 (1.61)	1.55 (1.60)
Depression	2.22 (0.61)	2.25 (0.61)	2.16 (0.60) ***	2.15 (0.59)	2.29 (0.63) ***

T-tests and ANOVA tests have been carried out to compare rural/urban and gender differences. *P < 0.05; **P < 0.01; ***P < 0.001.

**Table 2:** Ordinary least squares (OLS) model results: regress MED on depression for the whole sample (*N*=1,933).

	Covariates	Independent Variable	Whole
	Model 1	Model 2	Model 3
*Focal IV*	Estimates (SE)		
MED Consumption			
0 (Reference)			
1		-0.241 (0.055) ***	-0.212 (0.052) ***
2		-0.266 (0.052) ***	-0.181 (0.050) ***
3		-0.303 (0.058) ***	-0.193 (0.055) ***
Categorical			
Gender: Male	0.006 (0.033)		0.008 (0.033)
*Hukou*: Rural	0.040 (0.030)		0.044 (0.030)
Married/Partnered: Yes	-0.153 (0.029) ***		-0.154 (0.029) ***
Literate: Yes	-0.135 (0.030) ***		-0.132 (0.030) ***
Smoking: Yes	-0.041 (0.035)		-0.039 (0.035)
Drinking: Yes	-0.016 (0.034)		-0.014 (0.034)
Exercise: Yes	-0.061 (0.029) *		-0.058 (0.029) *
Social insurance: Yes	-0.063 (0.028) *		-0.062 (0.028) *
Income: Sufficient	-0.336 (0.038) ***		-0.323 (0.038) ***
Continuous			
Age	0.003 (0.002)		0.003 (0.002)
Life Satisfaction	-0.026 (0.018)		-0.029 (0.018)
Self-Rated Health	-0.071 (0.016) ***		-0.068 (0.016) ***
Number of Chronic Diseases	0.034 (0.008) ***		0.035 (0.008) ***

*P < 0.05; **P < 0.01; ***P < 0.001.

**Table 3:** Ordinary least squares (OLS) model results: regress MED on depression for the Subsamples.

	Rural	Urban	Male	Female
	Model 1	Model 2	Model 3	Model 4
*Focal IV*				
MED Consumption				
0 (Reference)				
1	-0.182 (0.064) **	-0.251 (0.093) **	-0.205 (0.079) **	-0.220 (0.071) **
2	-0.146 (0.061) *	-0.246 (0.085) **	-0.194 (0.074) **	-0.168 (0.068) *
3	-0.143 (0.070) *	-0.276 (0.092) **	-0.190 (0.081) *	-0.204 (0.078) **
Categorical				
Gender: Male	-0.003 (0.041)	0.029 (0.055)		
*Hukou*: Rural			0.040 (0.042)	0.046 (0.043)
Married/Partnered: Yes	-0.114 (0.036) **	-0.240 (0.053) ***	-0.169 (0.039) ***	-0.133 (0.044) **
Literate: Yes	-0.162 (0.037) ***	-0.074 (0.052)	-0.184 (0.043) ***	-0.088 (0.042) *
Smoking: Yes	-0.022 (0.043)	-0.075 (0.060)	-0.035 (0.038)	-0.070 (0.082)
Drinking: Yes	-0.016 (0.041)	-0.012 (0.059)	0.013 (0.038)	-0.096 (0.068)
Exercise: Yes	-0.065 (0.037) .	-0.048 (0.047)	-0.048 (0.039)	-0.061 (0.043)
Social insurance: Yes	-0.061 (0.034) .	-0.061 (0.051)	-0.070 (0.039) .	-0.052 (0.041)
Income: Sufficient	-0.351 (0.044) ***	-0.238 (0.080) **	-0.310 (0.053) ***	-0.331 (0.056) ***
Continuous				
Age	0.001 (0.002)	0.006 (0.003) .	0.002 (0.003)	0.005 (0.003) .
Life Satisfaction	-0.041 (0.022) .	-0.007 (0.033)	-0.033 (0.026)	-0.028 (0.026)
Self-Rated Health	-0.062 (0.019) **	-0.087 (0.029) **	-0.044 (0.022) .	-0.090 (0.023) ***
Number of Chronic Diseases	0.039 (0.011) ***	0.034 (0.013) *	0.024 (0.011) *	0.047 (0.012) ***

*P < 0.05; **P < 0.01; ***P < 0.001.

## 4 Results

The characteristics of the sample population are detailed in **Table 1**. Among the Chinese older adults surveyed, MED consumption was notably high, with only 8.21% reporting no intake of MED products. More than two-thirds of people held rural *Hukou* status, and the sample included a higher proportion of female respondents. Literacy was observed in more than half of the participants, and a significant majority (86.16%) perceived their family income as sufficient for daily needs. However, only a small fraction (31.23%) reported having social security or commercial insurance coverage. In terms of well-being, the respondents’ average life satisfaction was 3.65 out of 5, and their average self-rated health was 3.59 out of 5. On average, respondents reported 1.50 types of chronic diseases out of a possible 12. Depression levels within the population ranged from 1 to 5, with an average score of 2.22, suggesting a relatively low to medium level of depression.

The rural-urban comparison revealed a marked nutritional advantage among urban residents, evidenced by a significantly higher proportion (23.27%) of urban individuals consuming all three types of MED products, in contrast to only 15.22% among rural residents, suggesting that urban individuals were more likely to have a diversified diet including all three types of MED products. Urban residents exhibited advantages in literacy, income levels, and physical activity, as well as higher scores in life satisfaction and self-rated health. However, despite these nutritional benefits, the study also revealed a lower percentage of urban residents with social insurance coverage and a higher prevalence of chronic diseases among them, although rural older adults demonstrated significantly higher average depression scores. A similar pattern emerged in the male-female comparison, where females exhibited higher levels of depression and lower average MED consumption, alongside disadvantages in literacy, exercise, life satisfaction, and self-rated health. Nevertheless, females reported a higher rate of social insurance coverage and sufficient income, despite a greater incidence of chronic diseases.

**Figure 1** illustrates the dynamic relationship between MED consumption and depression levels across the study sample. In the overall sample (**Figure 1A**), a distinct downward trend in depression levels was observed as the number of MED types consumed increased. This pattern was consistently reflected across all four subsamples (**Figures 1B–E**). Notably, urban older adults exhibited a more pronounced reduction in depression levels compared to their rural counterparts in response to MED consumption. For rural residents, a significant decrease in depression levels was evident when transitioning from 0 to 1 type of MED consumption; however, depression levels remained relatively stable between 2- and 3-MED consumption. In contrast, urban older adults experienced a sharp decline in depression levels from 0 to 1 type of MED consumption, with further decreases observed at 2- and 3-MED consumption, reaching the lowest levels at 3-MED consumption. Moreover, gender subgroups (**Figures 1D, E**) revealed some disparities. For males, a consistent downward trend in depression levels was found as MED consumption increased, suggesting that males who consumed three types of MED products were the least likely to be depressed. Conversely, the female sample revealed that depression levels by 1- and 3-MED consumption were equally lower than that of 0-MED consumption, while female respondents who consumed two types of MED products even had higher depression levels compared to 1-/3-MED female consumers.

Ordinary Least Squares (OLS) model results are presented in **Tables 2**, **3**. **Table 2** presents three models: Model 1 illustrates the association between depression levels among older Chinese adults and so socio-demographic/health variables that are covariates; Model 2 focuses on the impact of MED consumption on depression levels; and Model 3 shows the results for the whole sample.

In Model 1, it was estimated that sufficient income for daily costs, literacy, marriage status, self-rated health, and number of chronic diseases were crucial factors that had a significant impact on depression levels. As for MED consumption, Model 2 revealed that the more types of MED products consumed by older Chinese adults were, the lower their depression levels became, indicating 3-MED consumption decreased the depression level by 0.303 units (compared with the reference group pf 0-MED consumption). Model 3 incorporates the effects of MED Consumption along with a range of covariates. By including covariates, Model 3 controls for potential confounding factors, providing a clearer estimate of the true relationship between MED Consumption and depression. Even after controlling for other variables, MED Consumption continues to show a significant negative relationship with depression. The strength of this relationship is slightly reduced compared to Model 2 (which includes only the independent variable), but it remains robust.

**Table 3** expanded the analysis by incorporating subsample variations, which accounted for rural/urban and male/female distinctions compared with the results of the whole sample. Urban areas showed stronger negative associations between MED consumption and depression across all levels. Notably, 1-MED consumption of rural residents was the most significant, reporting it decreased the depression level by 0.182 units. Conversely, in urban areas, 3-MED consumption proved to be the most significant, resulting in a decrease of 0.276 units in depression levels. However, the case was different in the gender subgroup. The consumption of 1-MED was the most significant factor in reducing depression levels for both males and females, with reported decreases of 0.205 and 0.220, respectively.

## 5 Nomenclature

Focusing on older adults in China, this study examined the association between Meat-Egg-Dairy (MED) product consumption and depressive symptoms, with a specific concentration on rural/urban differences and gender disparities. The findings revealed significant interplays between MED consumption and reduced depression levels, with notable variations across different demographic subgroups.

Overall, the data analysis demonstrated a consistent negative association between MED consumption and depression, which persisted even after controlling for various socio-demographic and health-related factors. It was revealed that compared to older adults in China who did not have MED product intake, individuals consuming MED exhibited significantly lower levels of depression. An interesting finding was that older adults in China exhibited relatively high levels of MED consumption, with male and urban respondents demonstrating significantly greater consumption. These disparities even extended across various sociodemographic and health factors. These findings aligned with previous literature, which suggested that males and urban residents were traditionally associated with higher protein intake, larger portion sizes, and increased meat consumption—patterns that tended to persist into old age (59, 61–63).

The observed inverse relationship between MED consumption and depression levels aligned with previous studies suggesting that dietary patterns rich in protein, essential fatty acids, and numerous micronutrients might play a protective role against mental disorders in older adults (64, 65). MED products have been proven to be rich sources of high-quality proteins, vitamins, zinc, and fatty acids, interacting with better psychological health outcomes (66, 67). However, while the results demonstrated a significant association, a direct causal relationship was not established. Individuals with better depressive symptom status were more likely to maintain a varied diet including MED products, rather than the consumption of these foods directly leading to improved depressive symptoms. The bi-directional interplay between diet and mental health has been noted in previous studies, and further investigations are needed in the context of Chinese older adults (68). This gap in establishing causality stemmed from several methodological challenges inherent to dietary studies: the complexity of dietary patterns that made it difficult to isolate the effects of specific food groups, the long-term nature of dietary influences on mental health, and the practical and ethical constraints of conducting randomized controlled trials with dietary interventions. Meanwhile, the relationship between MED consumption and depression might appear bidirectional, with several mechanisms potentially at play. Individuals with better mental health may be more likely to maintain varied diets, including MED products, while conversely, improved nutrition from MED consumption may support better mental health outcomes.

In the context of China, the findings revealed the disparities in MED consumption across rural and urban geographical settings influenced by the *Hukou* status of Chinese residents, with urban residents showing higher consumption levels. Recent data from the China Health and Nutrition Survey indicates that urban residents consumed on average 28.3% more meat compared to their rural counterparts, and urban residents had a greater mean intake of 30.9 g/d, compared to only 5.1 g/d in rural (69). These disparities were particularly pronounced in terms of healthcare access, aligning with the results from a meta-analysis that showed the mean pooled prevalence rate of depression in rural older Chinese is 29.2%, significantly higher than it was in urban older Chinese (20.5%) (70). Primarily, urban areas might have better access to nutrition, especially a broader variety and MED products of higher quality, potentially leading to greater nutritional advantages (71, 72). Urban environments typically offered a wider variety of food choices, including more Western-style products and imported foods. Supermarkets, specialty stores, and restaurants in cities provided easier access to a range of meat, egg, and dairy products. It might not be as readily available in rural areas, where a greater reliance on local markets and home-grown produce exists (73). Meanwhile, urban residents generally had higher incomes and education levels, which were associated with increased consumption of animal-source foods (74). Higher purchasing power allowed urban dwellers to afford more expensive food items, including high-quality meat and dairy products. Moreover, urban diets in China have undergone more rapid changes in recent decades, contributing to more pronounced effects of dietary shifts on health outcomes (75). The nutrition transition in China has been well-documented, with urban areas often leading the way in dietary changes, resulting in disparities in the dietary patterns and structures among rural/urban residents (76).

For gender differences in MED consumption among Chinese older adults, males were revealed to have higher MED consumption compared to females, with several factors contributing to this disparity. Genetically, physiological differences between genders, such as lower caloric requirements for women, might contribute to reduced MED consumption (77). Culturally, Chinese gender-specific dietary habits or beliefs about certain foods may influence consumption patterns (78). Traditional dietary habits in China have historically favored meat consumption among men, often associated with notions of masculinity and strength (79). This cultural preference may persist among older generations, influencing their food choices and consumption patterns. Societally, in many Chinese households, particularly among older generations, men often have more control over financial resources and decision-making regarding food purchases (80, 81). This economic power may allow them to prioritize their food preferences, including higher consumption of MED products. Conceptually, women, especially in older age groups, tend to be more health-conscious and may have greater nutrition knowledge compared to their male counterparts (82). This awareness may lead to more deliberate food choices, potentially limiting the consumption of certain MED products perceived as less healthy or associated with higher caloric intake.

The findings found in **Figure 1**, **Table 2** revealed a complex relationship. More types of MED product consumption were associated with lower depression scores, indicating a better mental health status. This suggests MED products might have a protective effect against depression, with other socio-demographical and health factors affecting the depressive symptoms in this population. Firstly, adequate protein intake, which is often associated with MED consumption, is crucial for maintaining muscle mass, cognitive function, and overall health in older adults (83). Improved physical health and functional status may, in turn, contribute to better mental health outcomes. Secondly, certain nutrients found in MED products, such as omega-3 fatty acids in fish, vitamin B12 in meat and dairy, and tryptophan in eggs, have been linked to improved mood and cognitive function (84). These nutrients play essential roles in neurotransmitter synthesis and brain health, potentially contributing to the observed protective effect against depression. Moreover, the social and cultural aspects of food consumption should not be overlooked. In Chinese culture, meat dishes often play a central role in social gatherings and festivals (85). The ability to consume and share these foods may contribute to a sense of social connection and cultural participation, which are important factors in maintaining a healthy mental state in older adults.

The Ordinary Least Squares (OLS) regression models provided valuable insights into the cross-sectional relationships between Meat, Egg, and Dairy (MED) consumption and depression scores among older Chinese adults. The observed associations could be bidirectional, with better mental health potentially leading to improved dietary habits, or unmeasured confounding factors influencing both MED consumption and depression scores. According to the results of **Tables 2**, **3**, the significant association between MED consumption and depression levels was influenced by various elements, and the strength of this association varied across different levels of MED consumption and subgroups. For instance, factors such as age-related health declines, loss of social roles, and changes in family dynamics may contribute to increased depression risk, regardless of dietary habits, which highlights the multifaceted nature of mental health in older adults (86). Excessive MED consumption may not provide additional benefits and could potentially have negative effects on mental health. Since the relationship between MED consumption and depression scores estimated by OLS models was non-linear, the potential adverse effects of high MED consumption on mental health might exist (87). These possible negative effects have been revealed in previous studies. Researchers indicated that higher meat consumption, particularly processed meat, was positively associated with inflammatory markers, potentially exacerbating depressive symptoms (88). Regarding egg consumption, a study found that the addition of half an egg per day was associated with more deaths from heart disease, cancer, and all causes (89). Meanwhile, high intakes of dairy products increased the risk for cancers, as men who consumed three or more servings of dairy products a day had a 141% higher risk for death due to prostate cancer compared to those who consumed less than one serving, while women who excessively consumed of cow’s milk per day had a 30% increased chance for breast cancer (90, 91). These relationships were further complicated by traditional Chinese dietary philosophy, which emphasizes balance and suggests that excessive consumption of any food category may disrupt physical and mental well-being. Age-related changes in digestion and metabolism may amplify these effects in older adults.

Meanwhile, specific attention should be paid to rural residents, since the impact of the types of MED consumption on their depression level was investigated to be less magnificent compared to urban residents. This finding may be related to the quality of MED products available in rural areas. While rural residents with high MED consumption may consume more of these foods, the nutritional quality may be lower compared to urban areas. Limited access to fresh, high-quality meat and dairy products in rural regions can result in the consumption of more processed or lower-quality alternatives, which may not provide the same nutritional benefits (92). Additionally, rural residents often engage in more physically demanding occupations and daily activities, such as farming or manual labor, potentially masking or increasing/reducing the observable effects of dietary factors on depressive symptoms (93). Moreover, the association between MED consumption and depression scores in rural areas may also be influenced by limited access to healthcare services, particularly mental health resources. Rural regions in China often face challenges in healthcare infrastructure and availability of specialized services, which may have potential interactions with diets and depressive symptom status (94).

Lastly, the findings indicating higher depression levels among female older adults have underscored the gender-specific dynamics influencing dietary habits and health outcomes. For instance, the data analysis revealed that the association between MED consumption and depression scores varied across different age groups in females, potentially reflecting the impact of hormonal changes throughout the lifespan. The relationship appeared to be strong in older women, coinciding with the perimenopausal and early postmenopausal periods. This finding suggests that hormonal fluctuations during menopause can influence both nutritional needs and mental health status, highlighting that adequate MED consumption is particularly important for maintaining mental well-being during hormonal transitions (95). Meanwhile, traditional beauty standards in Chinese culture often emphasize slimness, which is significantly associated with body image concerns potentially influencing dietary choices and attitudes toward food consumption (96). This awareness may persist to their older ages, potentially reflected in the increase in depression levels from 1- to 2-MED consumption. Additionally, since a considerable population of Chinese females are responsible for cooking, leading to differential dietary patterns. In prioritizing family preferences over personal dietary choices, females may consume higher amounts of meat, eggs, and dairy products, including fatty meats and dairy, which could exceed their digestive capacity. It is important to note that women who prepare meals may have greater awareness of their nutritional intake and may derive satisfaction from providing nourishing food for themselves and their families (97). Conversely, cooking responsibilities can also be a source of stress, particularly for older adults with physical limitations.

This study has several noteworthy limitations that warrant consideration. The reliance on self-reported data for dietary habits and physical assessments introduces potential measurement errors and recall bias, particularly given the complexity of accurately reporting food consumption patterns. Moreover, self-reported depressive symptoms may be influenced by social desirability bias, especially within Chinese culture where mental health stigma persists. The absence of objective measurements, such as biomarkers or clinical assessments, further compounds these methodological limitations. A significant constraint lay in the imbalanced rural/urban subsamples, with rural residents being overrepresented. This disproportion may reduce statistical power for detecting urban-specific associations and limit the generalizability of findings to urban populations. The study’s cross-sectional nature precludes establishing causal relationships between MED consumption and depressive symptoms, leaving temporal relationships unclear. The findings’ applicability to other cultural or geographical contexts may be limited due to China’s unique characteristics. These include distinct dietary patterns, cultural norms regarding mental health and aging, and healthcare system features. Additionally, seasonal variations in food availability and consumption patterns were not captured in our single-time-point assessment. Future research should address these limitations by incorporating objective dietary measurements, ensuring balanced rural-urban sampling, conducting longitudinal studies, and examining potential mediating factors. Such enhancements would strengthen the validity and generalizability of findings regarding the relationship between MED consumption and depressive symptoms among older adults.

## 6 Conclusions

Despite such limitations, this study provides important insights into the complex relationship between MED consumption and depressive symptoms among older adults in China. The findings highlight significant gender and urban-rural disparities in both dietary patterns and mental health outcomes. While moderate MED consumption appears to have a protective effect against depression, the relationship is non-linear and influenced by various sociodemographic and health-related factors. The results underscore the need for nuanced, targeted approaches to nutrition and mental health interventions for older adults in China. Policies and programs should consider the distinct needs and challenges faced by different subgroups, particularly rural residents and women. Future research should aim to address the limitations of this study by incorporating more detailed dietary assessments, objective measures of mental health, and a broader range of potential confounding factors. Ultimately, this research contributes to our understanding of the intricate connections between diet, demographic factors, and mental health in aging populations. As China continues to grapple with the challenges of an aging society and ongoing nutrition transition, these findings can inform evidence-based strategies to promote both physical and mental health among older adults.

## Data Availability

The original contributions presented in the study are included in the article/supplementary material. Further inquiries can be directed to the corresponding author.
